# Single-Stage Reconstruction of a Complex Full-Thickness Auricular Defect Following Excision of Rapidly Progressive Squamous Cell Carcinoma in a 100-Year-Old Patient: A Case Report

**DOI:** 10.7759/cureus.116603

**Published:** 2026-09-20

**Authors:** Ludmila Rodrigues Ribeiro da Silva

**Affiliations:** 1 Burn Reconstructive Surgery, Shriners Hospitals for Children, Galveston, USA

**Keywords:** antia-buch flap, auricular reconstruction, cutaneous squamous cell carcinoma (scc), ear neoplasm, elderly patient, full-thickness auricular defect, local flap, plastic and reconstructive surgery, postauricular flap, single-stage reconstruction

## Abstract

Auricular reconstruction following oncologic excision remains challenging because of the complex three-dimensional anatomy of the ear, limited local tissue reserve, and the need to preserve contour, projection, and external auditory canal patency. Large full-thickness defects involving multiple auricular subunits are particularly difficult to reconstruct without substantial alteration of auricular dimensions or the need for cartilage grafting or staged procedures.

This case describes a 100-year-old woman with a rapidly progressive auricular lesion involving the right helix, antihelix, and a portion of the conchal bowl. Serial clinical photographs obtained 19 days apart during the preoperative evaluation documented substantial interval progression of the lesion. Gross pathological examination documented a lesion measuring 4.4 × 3.9 cm within a surgical specimen measuring 5.5 × 4.4 × 2.4 cm. Histopathological examination confirmed an invasive, ulcerated, well-differentiated squamous cell carcinoma (SCC) with cartilage involvement and negative surgical margins. Following excision, a substantial full-thickness auricular defect involving multiple contiguous subunits remained. Immediate reconstruction was performed in a single stage using a three-component local tissue approach consisting of bidirectional advancement of the residual superior and inferior helical segments combined with anterior recruitment of posterior auricular skin. This strategy was designed to restore helical continuity, provide soft-tissue coverage, and preserve as much of the remaining native auricular framework as possible without cartilage grafting or a planned staged reconstruction.

Postoperative healing was uneventful, without flap necrosis, wound dehiscence, infection, or other major complications. At 76 days of follow-up, the reconstructed auricle demonstrated healed soft-tissue coverage, maintenance of overall auricular contour and projection, and external auditory canal patency. This case illustrates the feasibility of combining residual helical and posterior auricular tissues as a single-stage reconstructive option for selected complex full-thickness auricular defects when adequate viable local tissue remains. Longer follow-up and objective postoperative measurements are needed to assess long-term dimensional stability and durability.

## Introduction

The auricle is a frequent site of cutaneous malignancy because of its prominent anatomical position and cumulative exposure to ultraviolet radiation. Cutaneous squamous cell carcinoma (cSCC) is of particular concern in this region because tumors involving the ear have traditionally been associated with higher-risk clinical behavior, including locally aggressive growth and an increased potential for regional metastasis [1,2]. Achieving oncologic clearance is a primary objective; however, excision of tumors involving the cartilaginous framework may result in complex full-thickness defects that pose substantial reconstructive challenges.

Auricular reconstruction is technically demanding because the ear consists of a thin soft-tissue envelope overlying an intricate three-dimensional cartilaginous framework, with limited adjacent tissue available for redistribution. Successful reconstruction requires more than simple wound coverage and should aim to preserve or restore auricular height, projection, helical continuity, contour, and external auditory canal patency while minimizing visible scarring and donor-site morbidity [3-6]. Consequently, the reconstructive approach must be individualized according to the location, size, and depth of the defect; the extent of cartilage loss; the condition and mobility of the remaining auricular tissues; and patient-specific factors [3-6].

Reconstructive options range from secondary intention healing, primary closure, and grafting to local advancement or transposition flaps, cartilage-supported reconstruction, and staged procedures [3-6]. For full-thickness marginal helical defects, the Antia-Buch chondrocutaneous advancement flap mobilizes adjacent helical tissue toward the defect to restore rim continuity in a single stage [7-9]. The posterior auricular region provides an additional local tissue reservoir with a favorable color and texture match and a relatively concealed donor site [10-12]. Combining these local tissue sources may be particularly useful when a defect extends across multiple auricular subunits and advancement of the residual helix alone would create excessive tension, shortening, or distortion [13-15].

Large and complex auricular defects may alternatively require cartilage-supported reconstruction or staged tissue transfer, and the selection of a reconstructive strategy must balance restoration of form and function against the complexity and burden of additional procedures. Clinical series of auricular reconstruction following skin cancer excision emphasize the importance of tailoring reconstruction to the characteristics of the individual defect and patient [16]. This consideration may be particularly relevant in older patients, in whom single-stage reconstruction may reduce the burden of additional operative procedures when adequate local tissue is available [17].

Defects extending across the helix and antihelix and into adjacent subunits such as the conchal bowl present additional challenges because loss of both soft tissue and cartilage may compromise auricular contour and structural support [18,19]. When residual structural support is insufficient, cartilage grafting combined with postauricular tissue transfer represents an additional reconstructive option [20]. These considerations emphasize that complex defects involving multiple contiguous auricular subunits may require integration of more than one established reconstructive principle.

This report describes the single-stage reconstruction of a substantial full-thickness auricular defect following excision of a rapidly progressive SCC in a 100-year-old woman. The defect involved the helix, antihelix, and a portion of the conchal bowl and was reconstructed using a three-component local tissue strategy combining bidirectional advancement of the residual superior and inferior helical segments with anterior recruitment of posterior auricular skin. This case illustrates the complementary use of local tissue reservoirs to preserve the remaining native auricular framework, restore helical continuity, and provide soft-tissue coverage while avoiding cartilage grafting and a planned multistage reconstruction.

## Case presentation

A 100-year-old woman presented with a rapidly enlarging ulcerated lesion involving the right helix, antihelix, and a portion of the conchal bowl (Figures 1, 2), which was subsequently confirmed on definitive histopathological examination to represent invasive SCC. On examination, the lesion was extensive, ulcerated, and malodorous, with clinical evidence of full-thickness auricular involvement. No palpable preauricular, postauricular, or cervical lymphadenopathy was identified. No additional imaging-based metastatic evaluation, including CT of the neck or chest, was performed before surgery. Given the rapid clinical progression of the lesion, the patient proceeded to definitive surgical treatment following clinical assessment. Shortly before surgery, yellow drainage developed from the ulcerated lesion, raising concern for local bacterial contamination; therefore, oral ciprofloxacin 500 mg twice daily was administered empirically for seven days before definitive excision.

**Figure 1:**
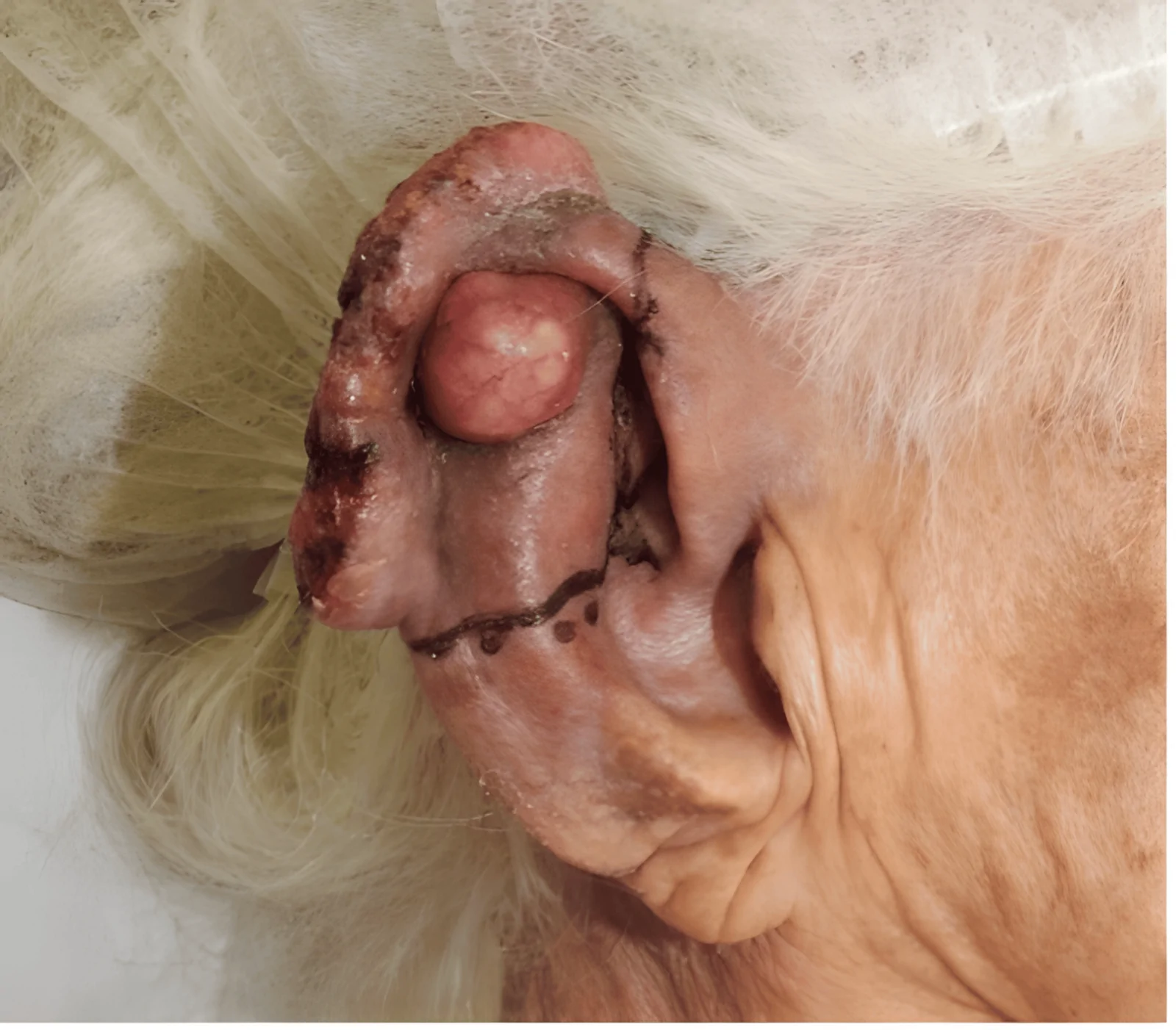
Preoperative right auricular squamous cell carcinoma involving the superior auricle Preoperative clinical photograph demonstrating an ulcerated exophytic squamous cell carcinoma involving the superior right auricle, with destruction of the helical rim and extension across adjacent auricular subunits. Surgical markings delineate the planned resection margins before oncologic excision and immediate reconstruction.

**Figure 2:**
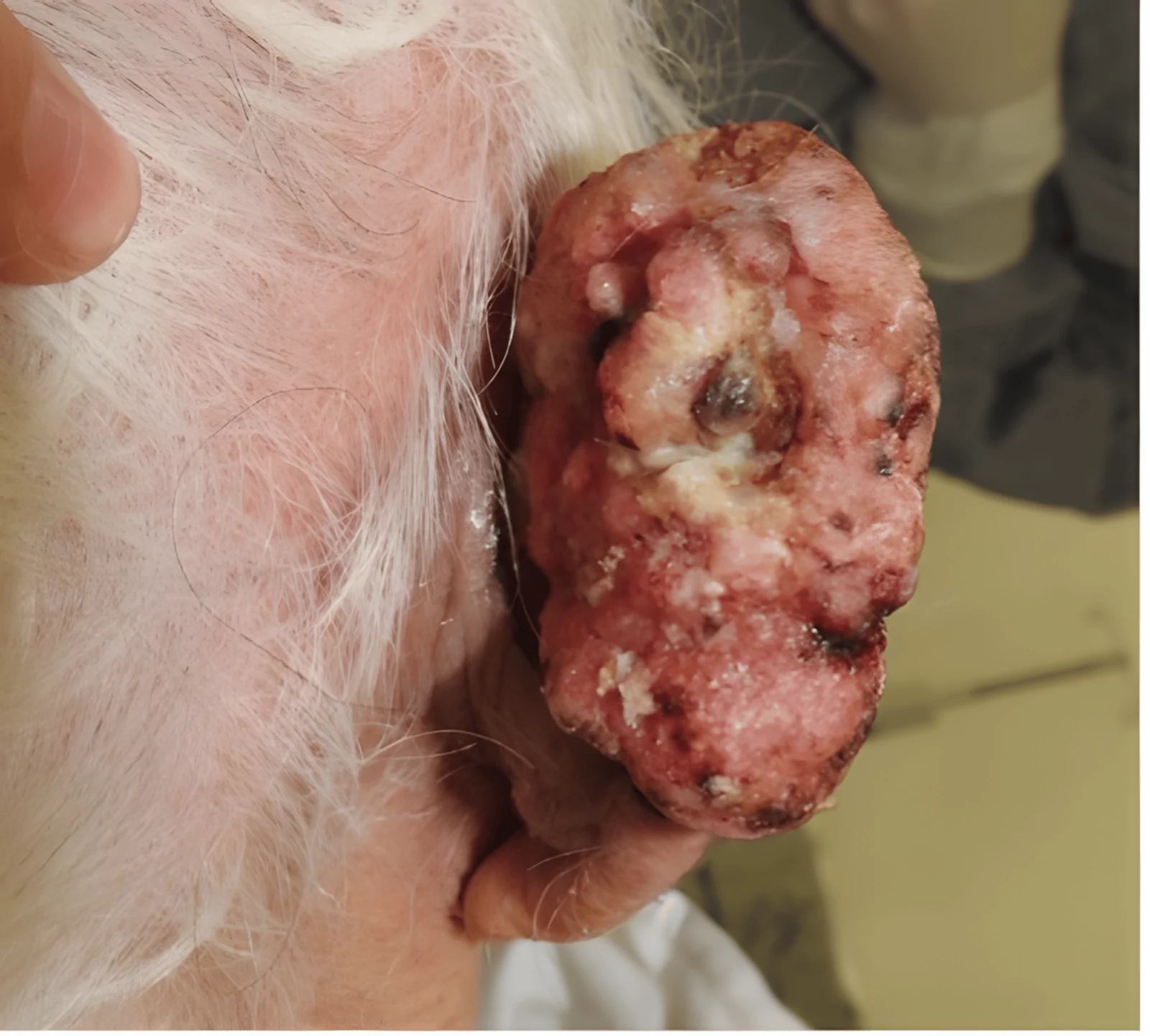
Posterior extent of the right auricular squamous cell carcinoma Posterior preoperative view demonstrating the extent of the lesion across the superior right auricle before oncologic excision. This perspective complements the anterior view by demonstrating the extent of tissue involvement relevant to planning resection and subsequent local tissue reconstruction.

During the preoperative evaluation and preparation for surgery, the lesion demonstrated substantial clinical progression over a short interval. Serial clinical photographs obtained 19 days apart documented progressive enlargement and further destruction of the involved auricular tissues (Figure 3). This rapid interval change, together with the full-thickness involvement of the auricle, reinforced the need for definitive oncologic excision.

**Figure 3:**
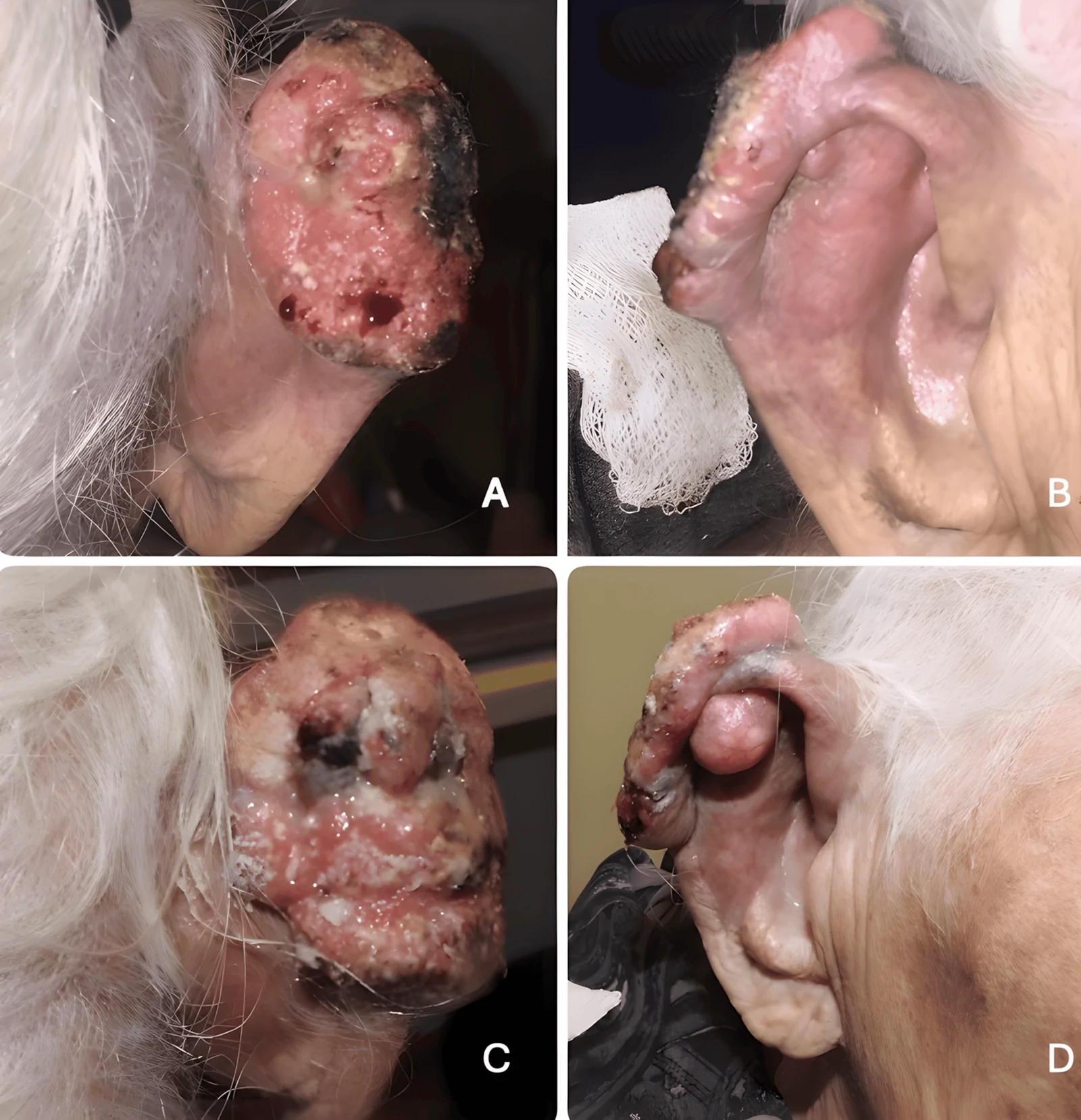
Rapid progression of right auricular squamous cell carcinoma over a 19-day preoperative interval Clinical photographs demonstrating progression of the auricular lesion over a 19-day interval while the patient was undergoing preoperative evaluation for surgical treatment. A, B: initial clinical appearance; C, D: appearance 19 days later, demonstrating interval progression of the lesion before surgery.

The lesion was excised under monitored sedation and local anesthesia with a surrounding margin of grossly uninvolved tissue to achieve oncologic clearance. Intraoperative frozen-section margin assessment was not performed; instead, the excised specimen was submitted to an external pathology laboratory for definitive histopathological evaluation. Gross examination demonstrated an irregular right auricular specimen measuring 5.5 × 4.4 × 2.4 cm containing a raised, grayish, irregular lesion measuring 4.4 × 3.9 cm. The larger specimen dimensions reflected excision of the tumor together with a surrounding margin of clinically uninvolved tissue rather than the dimensions of the tumor alone. Histopathological examination confirmed an invasive, ulcerated, well-differentiated SCC measuring 4.4 cm in greatest dimension, with infiltration of the auricular cartilage. No lymphovascular or perineural invasion was identified, and all surgical margins were negative for malignancy.

Histopathological processing and slide interpretation were performed by the external pathology laboratory. The treating institution retains the official pathology report but does not maintain the original histopathology slides or photomicrographs; consequently, these materials were unavailable for independent review or inclusion in this manuscript. The pathological findings reported in this case are therefore derived from the official pathology report.

Following excision, a substantial full-thickness defect remained, involving the helix, antihelix, and a portion of the conchal bowl. Because multiple contiguous auricular subunits and their cartilaginous framework were involved, direct approximation would have required substantial tissue mobilization, with the potential for excessive auricular shortening and distortion.

Given the patient's advanced age, a single-stage reconstruction using available local tissues was selected to minimize the operative burden of a multistage procedure while preserving as much of the native auricular framework as possible. The reconstruction incorporated three complementary local tissue components: the residual superior helical segment, which was advanced inferiorly; the residual inferior helical segment, which was advanced superiorly; and posterior auricular skin, which was recruited anteriorly to bridge the remaining central soft-tissue gap (Figure 4). This was not a V-Y advancement pattern; rather, the superior and inferior helical segments were advanced toward one another from opposite directions, with posterior auricular skin providing the third component of soft-tissue coverage.

**Figure 4:**
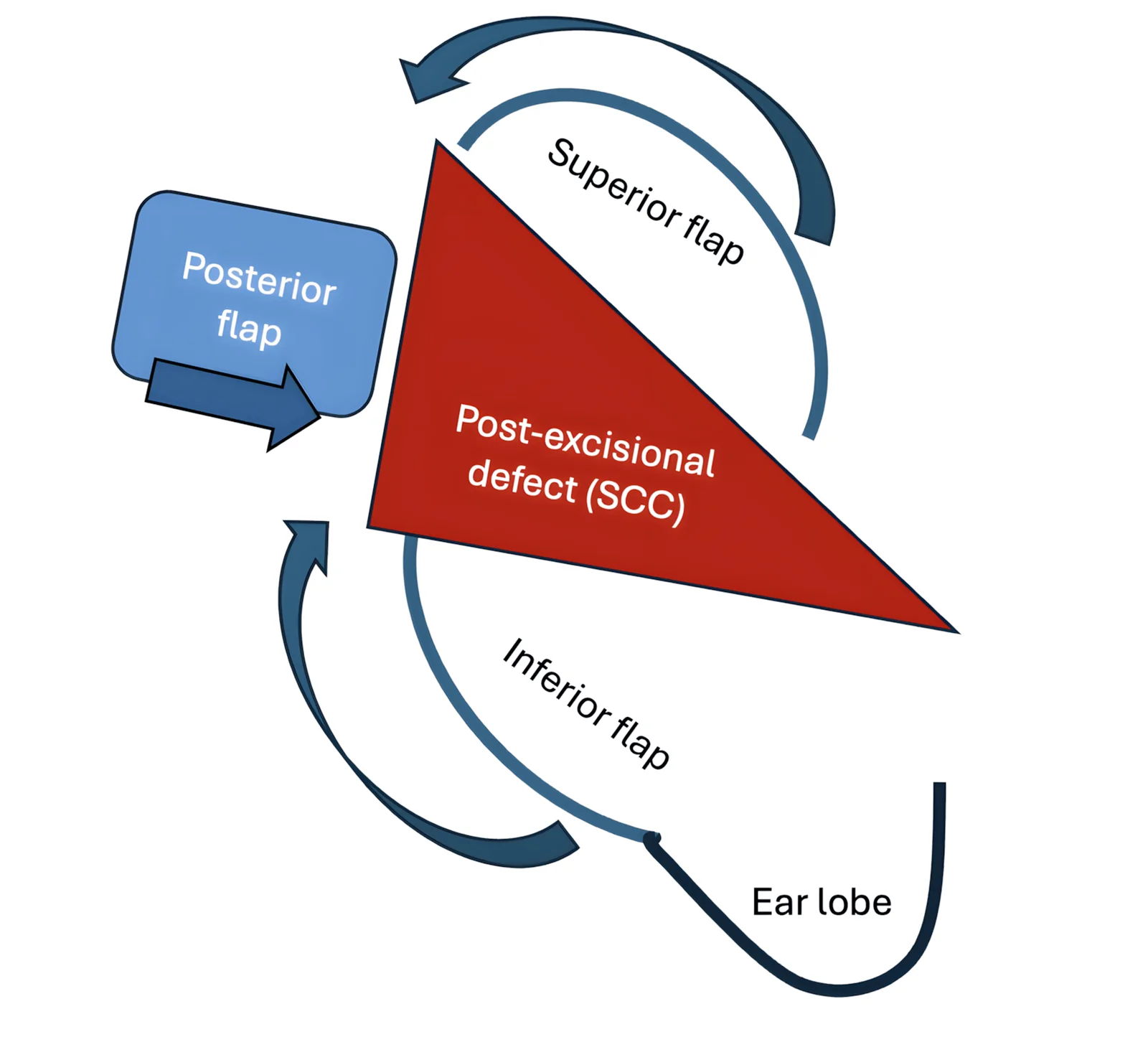
Schematic illustration of the three-component local tissue reconstruction Schematic representation of the single-stage reconstruction following oncologic excision. The red triangular area represents the post-excisional full-thickness defect, and the arrows indicate the direction of advancement of the remaining tissues. The residual superior helical segment was advanced inferiorly, the inferior helical segment was advanced superiorly, and posterior auricular skin was recruited anteriorly to bridge the remaining central soft-tissue gap. This was not a V-Y advancement pattern. The three complementary tissue components restored helical continuity and provided soft-tissue coverage while preserving as much of the remaining native auricular framework as possible. SCC: squamous cell carcinoma Image credit: Author, using Microsoft PowerPoint (Microsoft Corporation, Redmond, Washington, United States)

The residual superior helical segment was mobilized and advanced inferiorly, while the inferior helical segment was mobilized and advanced superiorly toward the central defect. This bidirectional advancement reduced the residual defect and re-established continuity of the helical rim. Posterior auricular skin was then mobilized and advanced anteriorly to bridge the remaining soft-tissue gap, where approximation of the superior and inferior helical components alone would have created excessive tension or additional auricular shortening.

The posterior auricular skin component functioned primarily as a soft-tissue bridge between the advanced superior and inferior auricular components. Because this component provided skin and soft-tissue coverage without replacing the resected cartilaginous framework, it was intended to bridge the residual defect rather than reproduce the native three-dimensional cartilaginous contour.

The three components were sequentially inset to restore helical continuity and provide soft-tissue coverage while maintaining external auditory canal patency and avoiding cartilage grafting or a staged interpolation procedure.

The immediate postoperative appearance demonstrated re-established continuity of the superior auricular rim and maintenance of external auditory canal patency (Figures 5, 6). Although some reduction in the dimensions of the reconstructed auricle was unavoidable because of the extent of oncologic resection, the use of opposing helical advancement combined with posterior auricular tissue allowed preservation of the remaining native ear without requiring cartilage grafting or staged reconstruction. 

**Figure 5:**
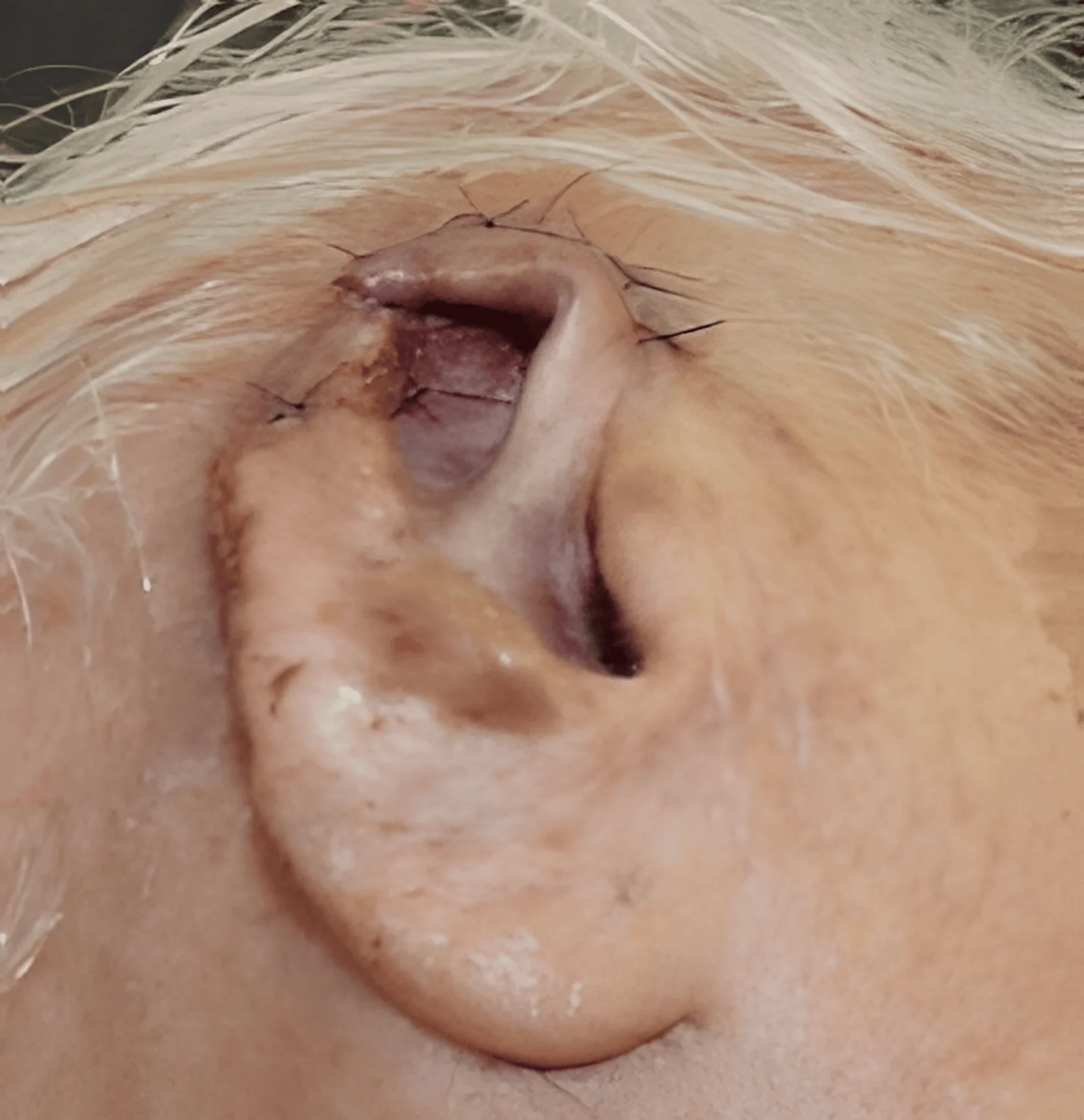
Immediate postoperative appearance following auricular reconstruction after squamous cell carcinoma excision Immediate postoperative lateral view of the reconstructed right auricle following oncologic excision and three-component local tissue reconstruction. The reconstructed helical rim and patent external auditory canal are visible.

**Figure 6:**
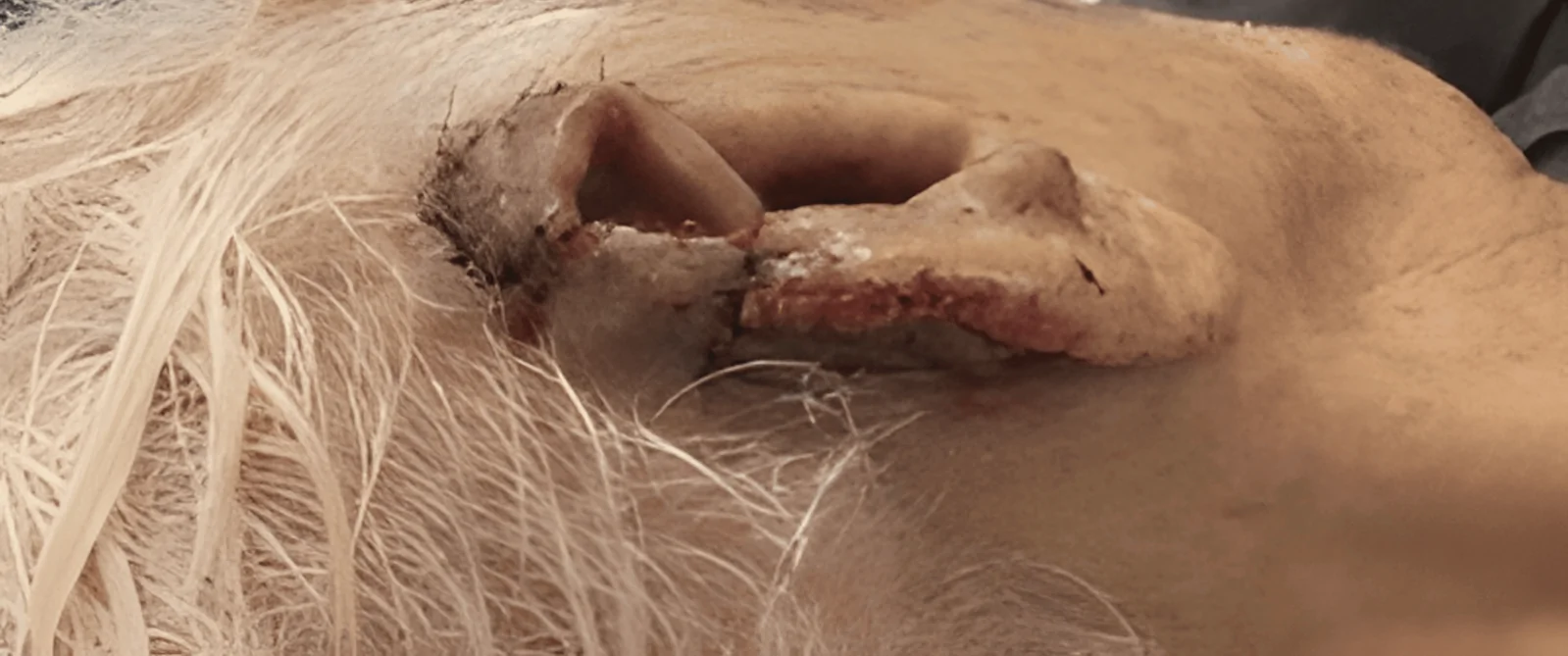
Immediate postoperative posterior view Immediate postoperative posterior view demonstrating the reconstructed right auricle and postauricular tissue recruitment.

Postoperatively, the patient healed without flap necrosis, wound dehiscence, hematoma, infection, or other major wound-related complications. At 76 days postoperatively, clinical examination demonstrated healed soft-tissue coverage, preservation of overall auricular contour and projection, and a patent external auditory canal (Figures 7, 8). Standardized postoperative auricular dimensions and validated patient-reported outcome measures were not obtained.

**Figure 7:**
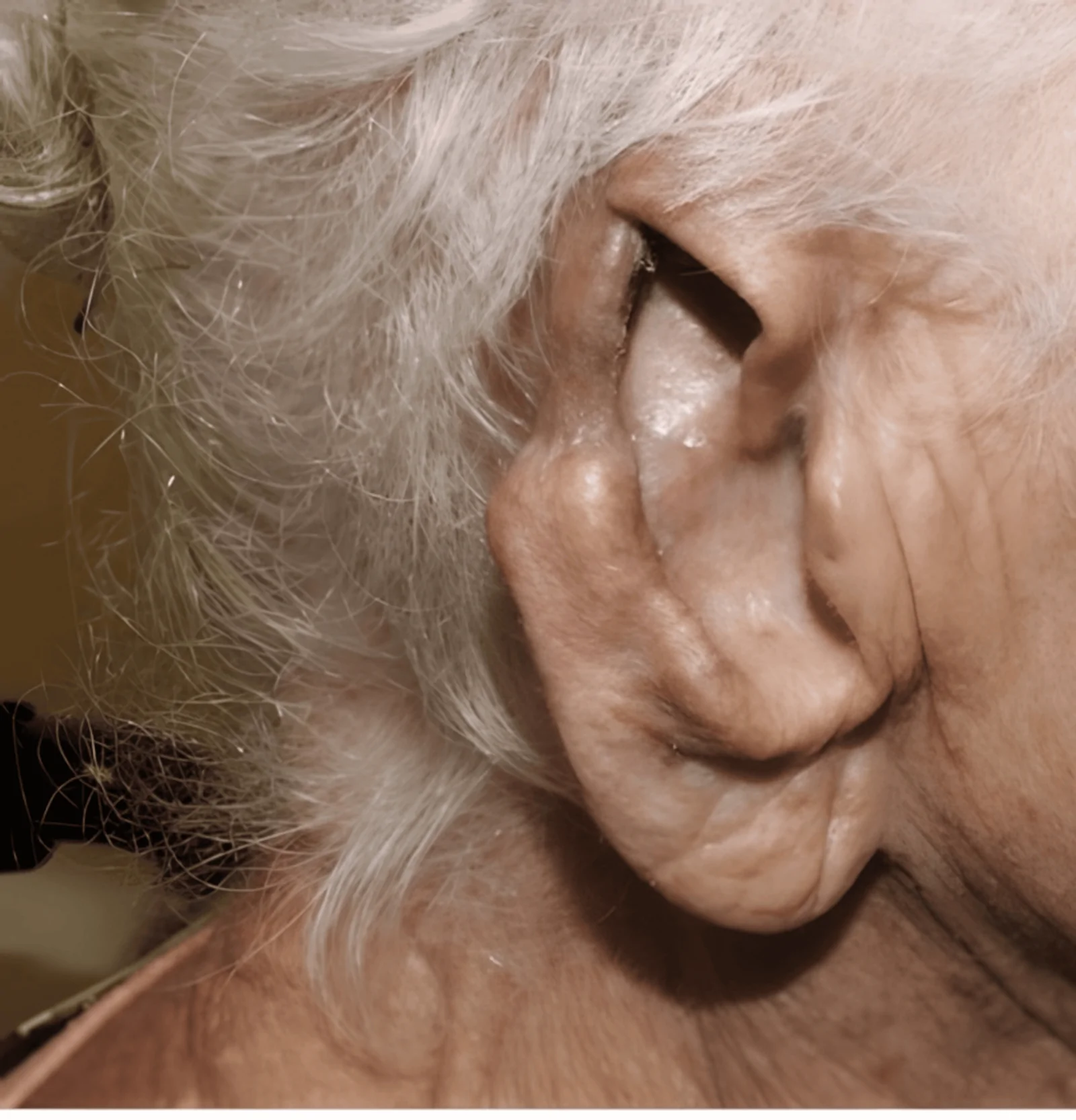
Postoperative lateral view (76-day) Lateral view obtained 76 days postoperatively demonstrating healed soft-tissue coverage of the reconstructed auricle and a patent external auditory canal, without clinically evident wound-related complications.

**Figure 8:**
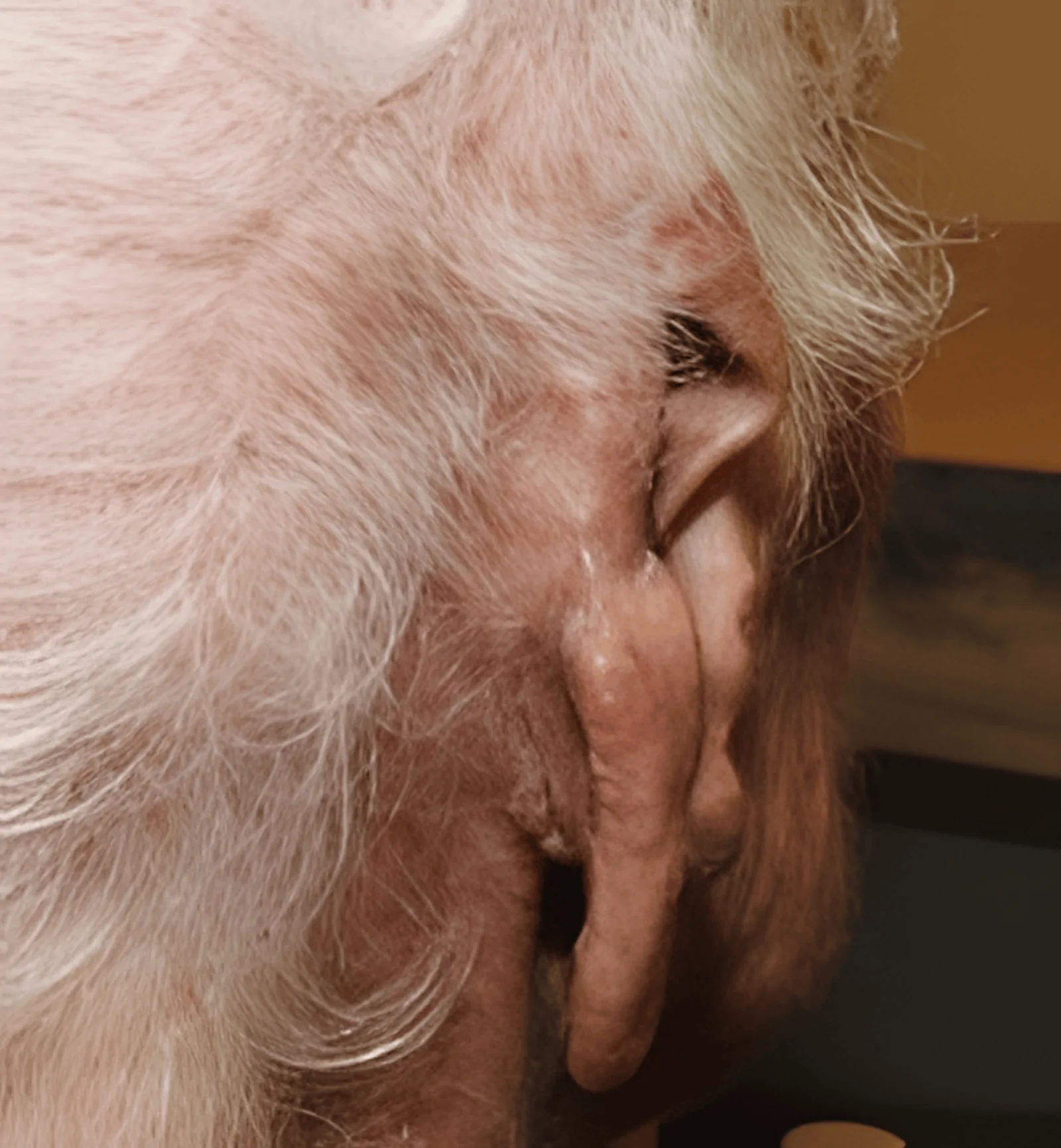
Postoperative posterior view (76-day) Posterior view obtained 76 days postoperatively demonstrating healed soft-tissue coverage following right auricular reconstruction, with preservation of overall auricular contour and no clinically evident wound-related complications.

## Discussion

Full-thickness auricular defects involving multiple contiguous anatomical subunits present a distinct reconstructive challenge because both soft-tissue coverage and preservation of the remaining three-dimensional cartilaginous framework must be considered. In the present case, oncologic excision resulted in a defect involving the helix, antihelix, and a portion of the conchal bowl. The distribution of tissue loss made simple approximation or isolated advancement of the residual helical rim less suitable because greater mobilization would have risked additional auricular shortening, tension, and distortion.

The Antia-Buch chondrocutaneous advancement flap remains a fundamental technique for reconstruction of full-thickness marginal helical defects. Superior and inferior helical segments are mobilized and advanced toward one another, allowing restoration of helical continuity with tissue of similar color, texture, and structural characteristics [7]. As defect size increases, however, the degree of advancement required may result in auricular shortening, alteration of normal proportions, or distortion of the reconstructed rim. Modifications of the original technique have therefore been developed to increase tissue mobility and extend its application to larger defects [8,9]. Lancer et al., in a series of 103 patients, demonstrated the versatility of a modified Antia-Buch approach for defects measuring up to 42 mm while emphasizing preservation of auricular dimensions as an important reconstructive objective [9]. In the present case, the defect extended beyond an isolated helical rim defect into the antihelix and conchal bowl; therefore, bidirectional helical advancement alone was considered insufficient to achieve closure without excessive tension or additional shortening.

The reconstruction was consequently conceived as a three-component local tissue strategy. The residual superior helical segment was advanced inferiorly, the inferior helical segment was advanced superiorly, and posterior auricular skin was recruited anteriorly to bridge the remaining central soft-tissue gap. This was not a V-Y advancement pattern. Distributing tissue recruitment among these three components reduced the degree of advancement required from the helical remnants and allowed greater preservation of the remaining native auricular framework.

The posterior auricular region is particularly useful in partial ear reconstruction because it provides a relatively generous local tissue reservoir with a favorable color and texture match and a donor site that can be largely concealed behind the ear [10-12]. Cordova et al. described the retroauricular skin as a “flap bank” for reconstruction of a variety of auricular defects [10], while Schonauer et al. demonstrated the versatility of posterior auricular flaps in partial ear reconstruction [11]. A systematic review by Worley and Cohen further demonstrated the broad applicability of the postauricular tissue reservoir through several flap designs for reconstruction of cutaneous auricular defects [12].

In the present reconstruction, the posterior auricular skin component functioned primarily as a soft-tissue bridge between the advanced superior and inferior auricular components. Because this component provided skin and soft-tissue coverage without replacing the resected cartilaginous framework, it was intended to bridge the residual defect rather than reproduce the native three-dimensional cartilaginous contour. Its addition reduced the amount of helical advancement and tension that would otherwise have been required to close the defect using the superior and inferior components alone.

Combining helical advancement with posterior auricular tissue recruitment is supported by previously described reconstructive principles. Zenn reported a single-stage technique combining helical advancement with a posterior auricular pull-through flap for combined helical and scaphal defects [13], and Goldberg et al. described postauricular cutaneous advancement for reconstruction of ear-rim defects [14]. Butler similarly demonstrated the usefulness of extended retroauricular advancement in a complex full-thickness auricular defect [15]. Therefore, the reconstruction described here should not be interpreted as an entirely new flap; rather, it represents patient-specific integration of established local tissue principles according to the distribution of the residual viable auricular tissue after oncologic excision.

Alternative reconstructive strategies could have been considered depending on the extent of structural loss. Defects with insufficient residual cartilaginous support may require cartilage grafting combined with local or postauricular flap coverage, while some postauricular techniques require a subsequent procedure for pedicle division and final inset [15,18-20]. Defects extending into the conchal bowl introduce additional anatomical considerations, and several reconstructive approaches have been described without a single technique demonstrating universal superiority [19]. In this patient, sufficient viable native auricular framework remained after excision to permit reconstruction using local tissues alone, avoiding cartilage grafting and a planned secondary reconstructive procedure.

Patient-specific considerations were particularly relevant because the patient was 100 years old. Chronologic age alone should not preclude meaningful auricular reconstruction, but the anticipated reconstructive benefit must be balanced against operative burden and the need for additional procedures. A large clinical series of auricular reconstruction following skin cancer excision supports individualized selection of reconstructive techniques [16]. Bolletta et al. specifically evaluated single-stage ear reconstruction following skin cancer excision in elderly patients and reported favorable outcomes with a low complication rate and no requirement for secondary reconstructive procedures in their series [17]. In the present patient, advanced age, adequate residual viable local tissue, and the feasibility of immediate local reconstruction favored a single-stage approach.

Oncologic considerations remained central to management. Serial clinical photographs obtained 19 days apart demonstrated substantial interval progression of the lesion. Definitive histopathological examination demonstrated a 4.4-cm invasive, ulcerated, well-differentiated SCC with auricular cartilage involvement. No lymphovascular or perineural invasion was identified, and all surgical margins were negative. Intraoperative frozen-section margin assessment can provide immediate evaluation of surgical margins and permit additional resection during the same procedure when a margin is involved. Frozen-section assessment was not performed in this case; instead, the excised specimen underwent definitive histopathological evaluation by an external pathology laboratory, which subsequently confirmed negative margins.

Auricular cSCC has been associated with higher-risk clinical behavior and an increased potential for regional metastasis [1,2]. Clinical assessment of the regional lymph node basins revealed no palpable preauricular, postauricular, or cervical lymphadenopathy. No additional imaging-based metastatic evaluation, including CT of the neck or chest, was performed before surgery. Given the auricular location, pathological tumor dimension of 4.4 cm, ulceration, and subsequently confirmed cartilage invasion, additional imaging-based staging may have provided further assessment of regional disease. The absence of imaging-based metastatic evaluation therefore limits assessment of regional or distant disease in this report.

At 76 days postoperatively, the reconstruction demonstrated healed soft-tissue coverage, preservation of overall auricular contour and projection, and external auditory canal patency, without flap necrosis, wound dehiscence, infection, or other major wound-related complications. These findings represent an early clinical outcome rather than evidence of long-term dimensional stability or durability.

Several limitations should be acknowledged. This is a single-patient retrospective case report with a 76-day postoperative follow-up, which represents an early reconstructive outcome and does not establish long-term dimensional stability, durability, local oncologic control, or regional disease status. Standardized preoperative and postoperative auricular measurements were not obtained, and the reconstructed auricle was not objectively compared with the contralateral ear; therefore, changes in auricular height, width, projection, or symmetry resulting from tissue advancement cannot be quantitatively determined. Validated patient-reported outcome or scar-assessment measures were also not prospectively collected. Histopathological processing and slide interpretation were performed by an external pathology laboratory, and the original slides and photomicrographs were unavailable for independent review; consequently, the pathological findings reported here are derived from the official pathology report. The absence of imaging-based metastatic evaluation also limits assessment of regional or distant disease. In addition, the serial preoperative photographs were not obtained with a standardized metric reference, precluding reliable retrospective calculation of a quantitative tumor growth rate. Finally, the specific distribution and viability of the residual auricular tissues after oncologic excision were favorable for this reconstruction and may not be reproducible in defects with greater cartilage loss or different anatomical distributions.

Despite these limitations, this case illustrates the feasibility of integrating bidirectional helical advancement with posterior auricular tissue recruitment in a single-stage reconstruction of a complex full-thickness defect involving multiple auricular subunits. The principal value of the approach lies not in establishing superiority over existing techniques but in demonstrating how complementary local tissue reservoirs may be combined when sufficient viable residual tissue remains and minimizing additional operative stages is desirable. Additional cases with longer follow-up, standardized dimensional assessment, and patient-reported outcomes are needed to evaluate reproducibility and long-term results.

## Conclusions

This case demonstrates the feasibility of a single-stage reconstructive approach using complementary residual helical and posterior auricular tissues for a complex full-thickness auricular defect. At 76 days of follow-up, the reconstruction demonstrated healed soft-tissue coverage, preservation of overall auricular contour and projection, and external auditory canal patency, without major postoperative complications. However, the short follow-up and absence of standardized quantitative auricular measurements limit assessment of long-term dimensional stability and durability. In selected patients with adequate viable residual tissue, this approach may represent a practical reconstructive option that avoids a planned staged procedure or cartilage grafting. Additional cases with longer follow-up and objective outcome measurements are needed to assess reproducibility and long-term results.
